# Synthesis and characterization of a *tert-*butyl ester-substituted titanocene dichloride: ^*t*-BuOOC^Cp_2_TiCl_2_

**DOI:** 10.1107/S2056989020011834

**Published:** 2020-09-04

**Authors:** Jackson S. McCarthy, Colin D. McMillen, Jared A. Pienkos, Paul S. Wagenknecht

**Affiliations:** a Department of Chemistry, Furman University, 3300 Poinsett Highway, Greenville, SC 29613, USA; b Department of Chemistry, Hunter Laboratories, Clemson University, Clemson, SC 29634, USA

**Keywords:** crystal structure, titanocene, carboxyl­ate anchoring group, metallocene

## Abstract

Bis[η^5^-(*tert*-butoxyca­rbonyl)­cyclo­penta­dien­yl]di­chlorido­titanium(IV), [Ti(C_10_H_13_O_2_)_2_Cl_2_], was synthesized from LiCp^COO*t*-Bu^ using TiCl_4_, and was characterized by single-crystal X-ray diffraction and ^1^H NMR spectroscopy. The distorted tetra­hedral geometry about the central titanium atom is relatively unchanged compared to Cp_2_TiCl_2_. The complex exhibits elongation of the titanium–cyclo­penta­dienyl centroid distances [2.074 (3) and 2.070 (3) Å] and a contraction of the titanium–chlorine bond lengths [2.3222 (10) Å and 2.3423 (10) Å] relative to Cp_2_TiCl_2_. The dihedral angle formed by the planes of the Cp rings [52.56 (13)°] is smaller than seen in Cp_2_TiCl_2_. Both ester groups extend from the same side of the Cp rings, and occur on the same side of the complex as the chlorido ligands. The complex may serve as a convenient synthon for titanocene complexes with carboxyl­ate anchoring groups for binding to metal oxide substrates.

## Abstract 2

The synthesis and characterization of a new titanocene dichloride complex with *tert-*butyl esters appended to the cyclo­penta­diene rings is reported.

## Chemical context   

Mol­ecules exhibiting charge-separated excited states have been shown to be useful in photocatalysis (Prier *et al.*, 2013 ▸), dye-sensitized photoelectrochemical cells (Hammarström, 2015 ▸; Kalyanasundaram & Grätzel, 1998 ▸) and dye-sensitized solar cells (DSSCs) (Ji *et al.*, 2018 ▸; Kalyanasundaram & Grätzel, 1998 ▸). One architecture used in compounds of this type is the Donor–π bridge–Acceptor (*D*–π–*A*) architecture, where absorption of a photon results in the transfer of charge from an electron-rich donor portion of the mol­ecule to an electron-poor acceptor portion through a conjugated π-linkage (Ji *et al.*, 2018 ▸). Alkynyl titanocenes utilizing titanocene acceptors and ferrocenyl or aryl­amine donors are promising candidates for sensitizers in DSSCs (Turlington *et al.*, 2016 ▸; Pienkos *et al.*, 2016 ▸, 2018 ▸; Livshits *et al.*, 2019 ▸). In photovoltaic technologies, the sensitizer must be attached to a semiconductor substrate, commonly TiO_2_, using an anchoring group exhibiting a high binding affinity for the substrate (Zhang & Cole, 2015 ▸; Kalyanasundaram & Grätzel, 1998 ▸). The most common anchoring group used with TiO_2_ semiconductors is the carboxyl­ate, chosen for its strong binding and conjugated π-electron system (Galoppini, 2004 ▸). Anchoring groups with conjugated π systems allow for improved device efficiency in DSSCs compared to anchoring groups with aliphatic or unconjugated linkages (Zhang & Cole, 2015 ▸). In alkynyl titanocene sensitizers, the alkyn­yl–titanium bond is sensitive to acid hydrolysis. As a result, the carboxyl­ate anchor must be masked with a protecting group to avoid carb­oxy­lic acid inter­mediates. Our research group has focused primarily on the *tert*-butyl protecting group, because *t*-butyl esters are relatively stable and have well documented deprotection strategies under mild conditions (Jung & Lyster, 1977 ▸; Theodorou *et al.*, 2018 ▸; Shaw *et al.*, 2008 ▸). Herein, we report the synthesis, crystallization, and structural analysis of a *t*-butyl ester substituted titanocene dichloride that will serve as a convenient synthon for *D*–π–*A* titanocenes with carboxyl­ate anchoring groups.
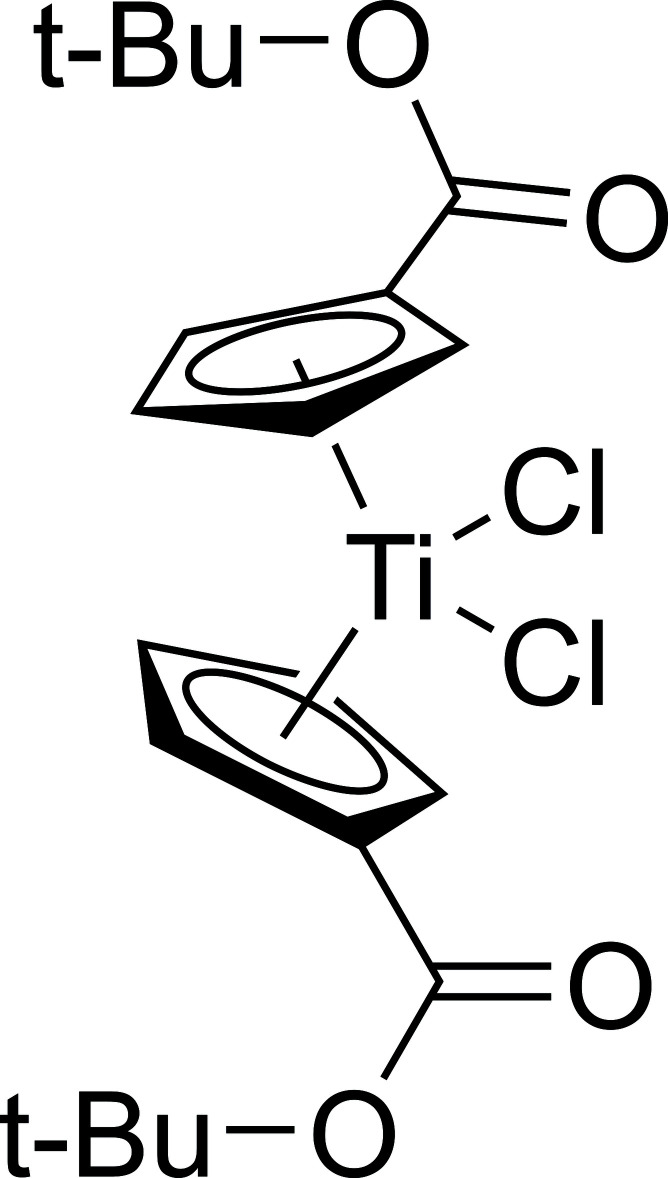


## Structural commentary   

**Figure 1:**
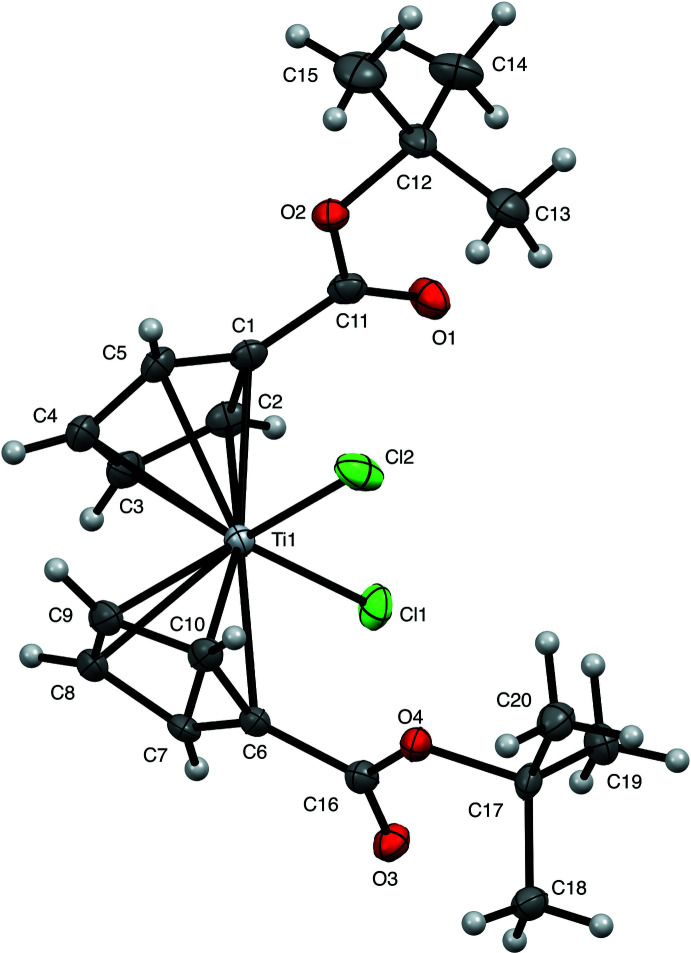
Structure of ^*t*-BuOOC^Cp_2_TiCl_2_ shown as 50% probability ellipsoids, with H atoms as small arbitrary spheres.

**Figure 2:**
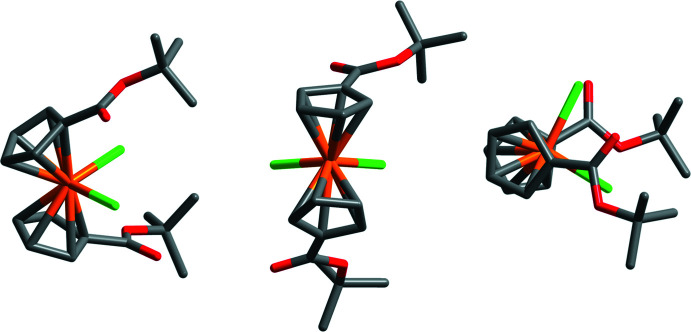
Ligand orientation in the structure of ^*t*-BuOOC^Cp_2_TiCl_2_.

While many titanocene and metallocene compounds have been characterized by X-ray diffraction, structures of ester-substituted metallocenes are comparatively rare. Here we present the structure of the *t*-butyl ester-substituted complex ^*t*-BuOOC^Cp_2_TiCl_2_ (Fig. 1 ▸). Though the data pool is small, our findings follow trends seen in previously reported structures. In the metallocenes of both vanadium and titanium, the addition of the ester shortens the metal–chlorine bond length by 0.02-0.04 Å [2.3222 (10) and 2.3423 (10) Å in the present titanocene] compared to the parent Cp_2_VCl_2_ (Tzavellas *et al.*, 1996 ▸) and Cp_2_TiCl_2_ (Clearfield *et al.*, 1975 ▸) complexes. A similar *M*—Cl bond contraction was not observed in the recent report of a titanocene with a bulky alkyl substituent appended to the Cp ring, (CpC(CH_3_)_2_CH_2_CH(CH_3_)_2_)_2_TiCl_2_ (Ceballos-Torres *et al.*, 2019 ▸), suggesting that the change is likely due to the inter­play of the electron-withdrawing nature of the ester and the π-donor character of the chlorido ligand. Furthermore, substitution at the Cp ring results in a slight elongation of the titanium–cyclo­penta­diene centroid distance [2.070 (3) and 2.074 (3) Å] by 0.011 to 0.015 Å in the ester-substituted titanocene here and as much as 0.016 Å in the alkyl substituted titanocene (Ceballos-Torres *et al.*, 2019 ▸). However, this trend is not noticeable between ester-substituted and unsubstituted vanadocene dichloride (Klepalová *et al.*, 2013 ▸; Tzavellas *et al.*, 1996 ▸). Substitution of esters at the Cp ring has little effect on the bond angles formed about the central metal in both titanium and vanadium compounds, with a centroid—Ti—centroid angle of 129.90 (12)° and a Cl—Ti—Cl angle of 95.23 (4)° observed here. In titanocenes, substitution at the Cp ring results in a decrease of the dihedral angle formed between the planes of the two Cp rings. This angle is 58.5° in titanocene dichloride (Clearfield *et al.*, 1975 ▸), but is 52.56 (13)° in this titanocene and 52.2° in the alkyl substituted titanocene (Ceballos-Torres *et al.*, 2019 ▸). This trend is not observed between substituted and unsubstituted vanadocene dichloride, where the dihedral angle is approximately 48° for both (Tzavellas *et al.*, 1996 ▸; Klepalová *et al.*, 2013 ▸). The dihedral angle formed between the esters and their associated Cp rings differs more in the titanocene than in other ester-substituted metallocenes. In ^*t*-BuOOC^Cp_2_TiCl_2_, these two angles are 8.2 (6)° and 15.7 (3)°. In the other ester substituted metallocenes, the angles differ by less than a degree (18.37 and 18.37° in ^PhOOC^Cp_2_VCl_2_ and 10.78 and 11.36° in ^PhOOC^Cp_2_NbCl_2_) (Klepalová *et al.*, 2013 ▸). The appended esters in ^*t*-BuOOC^Cp_2_TiCl_2_ extend from the same sides of both Cp rings, and occur on the same side of the complex as the chlorido ligands (Fig. 2 ▸). This is a similar arrangement to what occurs in ^EtOOC^Cp_2_NbBr_2_ and ^MeOOC^Cp_2_NbBr_2_·CH_2_Cl_2_, but differs from ^PhOOC^Cp_2_VCl_2_, ^PhOOC^Cp_2_NbCl_2_, and ^MeOOC^Cp_2_NbBr_2_, where the substituting esters are on opposing sides of their respective Cp rings, and also do not overlap with the halides (Klepalova *et al.*, 2013 ▸).

## Supra­molecular features   

**Figure 3:**
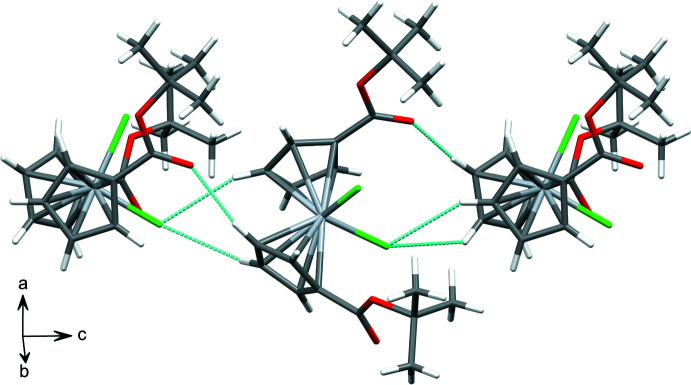
Chains of ^*t*-BuOOC^Cp_2_TiCl_2_ propagating along the *c* axis. Close contacts are depicted as dotted lines.

**Figure 4:**
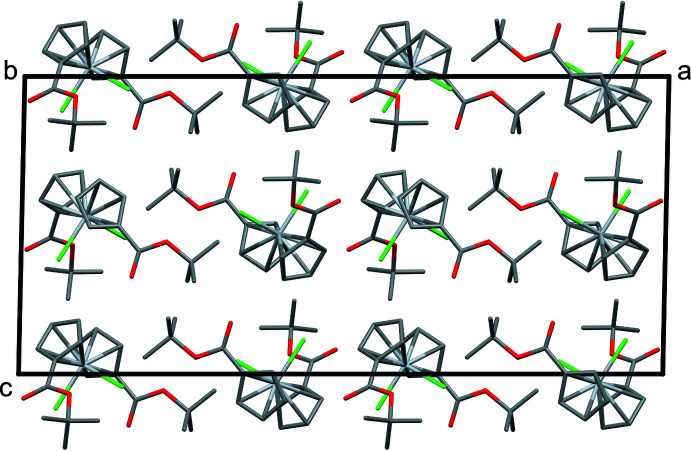
Packing of mol­ecules viewed along the *b* axis.

Inter­molecular contact geometries are shown in Table 1 ▸. Neighboring mol­ecules are connected along the *c*-axis direction *via* C9—H9⋯O1, C8—H8⋯Cl1, and C4—H4⋯Cl1 inter­actions to form chains (Fig. 3 ▸). Both Cp groups are angled toward the neighboring chlorine atom to enable these inter­actions. Neighboring mol­ecules along the *a*-axis are connected in a dimerized fashion *via* C7—H7⋯Cl1 inter­actions. The resulting packing diagram is shown in Fig. 4 ▸.

## Database survey   

A CSD search revealed nearly 200 hits for metallocene dichloride complexes, where the two cyclo­penta­diene ligands were monosubstituted (CSD Version 5.41, Update 2, May 2020; Groom *et al.*, 2016 ▸). Of these, only two, CSD entries CICPIP (vanadium) and CICPOV (niobium) are substituted by a protected carboxyl­ate (Klepalová *et al.*, 2013 ▸). Both of these utilize a phenyl-protecting group, and the carboxyl­ate carbon is bound to the Cp ring, similar to the *tert*-butyl-protected titanocene of the present study. Methyl- and ethyl-protected carboxyl­ate-substituted Cp ligands are reported in the niobium dibromide complexes CICPUB, CICQAI, and CICQEM (Klepalová *et al.*, 2013 ▸).

## Synthesis and crystallization   

Lithium *tert*-butyl ester cyclo­penta­dienide (Shaw *et al.*, 2008 ▸) (2.0278 g, 11.78 mmol, 1 eq) was dissolved by the addition of THF (15 mL) under an argon atmosphere. The reaction solution was chilled to 195 K and 1 *M* TiCl_4_ solution in toluene (6 ml, 6 mmol, 0.5 eq) was added *via* syringe. The solution changed from pale yellow to red–brown. After 5 minutes, the reaction was allowed to gradually warm to room temperature and stirred overnight. Solid impurities were filtered from the reaction mixture and the solvent was removed from the filtrate. Pentane (5 mL) was added, the mixture was filtered, and the solid impurities were washed with pentane and toluene. The solvent was removed from the filtrate and the resulting red porous solid was dissolved in CH_2_Cl_2_ (3 mL), and pentane (50 mL) was added to the solution. The solution was filtered, and the filtrate immediately began to form a precipitate in the filter flask. The resulting suspension was filtered yielding a red–orange powder (0.2823 g, 5.3% yield). ^1^H NMR (400 MHz, C_6_D_6_) δ 6.95 (2H), 6.04 (2H), 1.42 (9H).

Single crystals suitable for X-ray analysis were grown by slow evaporation of a hexa­nes solution of the crude product, following the removal of solid impurities. The mixture was chilled to 243 K to encourage further crystallization.

## Refinement   

Crystal data, data collection and structure refinement details are summarized in Table 2 ▸. Hydrogen atoms were placed in calculated positions using riding models, with C—H = 0.95 Å and *U*_iso_(H) = 1.2*U*_eq_(C) for aromatic hydrogen atoms, and C—H = 0.98 Å with *U*_iso_(H) = 1.5*U*_eq_(C) for methyl hydrogen atoms.

## Supplementary Material

Crystal structure: contains datablock(s) I. DOI: 10.1107/S2056989020011834/pk2643sup1.cif

Structure factors: contains datablock(s) I. DOI: 10.1107/S2056989020011834/pk2643Isup3.hkl

CCDC reference: 2025817

Additional supporting information:  crystallographic information; 3D view; checkCIF report

## Figures and Tables

**Table 1:** Hydrogen-bond geometry (Å, °)

*D*—H⋯*A*	*D*—H	H⋯*A*	*D*⋯*A*	*D*—H⋯*A*
C4—H4⋯Cl1^i^	0.95	2.78	3.632 (4)	149
C7—H7⋯Cl1^ii^	0.95	2.80	3.511 (4)	132
C8—H8⋯Cl1^i^	0.95	2.76	3.521 (3)	137
C9—H9⋯O1^i^	0.95	2.30	3.245 (4)	170

Symmetry codes: (i) 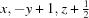; (ii) 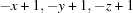.

**Table 2:** Experimental details

Crystal data	
Chemical formula	[Ti(C_10_H_13_O_2_)_2_Cl_2_]
*M*_r_	449.21
Crystal system, space group	Monoclinic, *C*2/*c*
Temperature (K)	100
*a*, *b*, *c* (Å)	29.3802 (19), 10.8106 (7), 13.6002 (9)
β (°)	91.214 (3)
*V* (Å^3^)	4318.7 (5)
*Z*	8
Radiation type	Mo *K*α
μ (mm^−1^)	0.67
Crystal size (mm)	0.21 × 0.04 × 0.04

Data collection	
Diffractometer	Bruker D8 Venture Photon 2
Absorption correction	Multi-scan (*SADABS*; Bruker, 2016 ▸)
*T*_min_, *T*_max_	0.923, 1.000
No. of measured, independent and observed [*I* > 2σ(*I*)] reflections	18758, 4007, 3053
*R*_int_	0.057
(sin θ/λ)_max_ (Å^−1^)	0.606

Refinement	
*R*[*F* ^2^ > 2σ(*F* ^2^)], *wR*(*F* ^2^), *S*	0.048, 0.106, 1.16
No. of reflections	4007
No. of parameters	250
H-atom treatment	H-atom parameters constrained
Δρ_max_, Δρ_min_ (e Å^−3^)	0.43, −0.40

Computer programs: *APEX3* (Bruker, 2017 ▸), *SAINT* (Bruker, 2013 ▸), *SHELXT2016* (Sheldrick, 2015*a* ▸), *SHELXL2016* (Sheldrick, 2015*b* ▸), *Mercury* (Macrae *et al.*, 2020 ▸) and *SHELXTL* (Sheldrick, 2008 ▸).

## supplementary crystallographic information

### Crystal data

**Table:**

[Ti(C_10_H_13_O_2_)_2_Cl_2_]	*F*(000) = 1872
*M**_r_* = 449.21	*D*_x_ = 1.382 Mg m^−^^3^
Monoclinic, *C*2/*c*	Mo *K*α radiation, λ = 0.71073 Å
*a* = 29.3802 (19) Å	Cell parameters from 8394 reflections
*b* = 10.8106 (7) Å	θ = 2.5–27.5°
*c* = 13.6002 (9) Å	µ = 0.67 mm^−^^1^
β = 91.214 (3)°	*T* = 100 K
*V* = 4318.7 (5) Å^3^	Column, orange
*Z* = 8	0.21 × 0.04 × 0.04 mm

### Data collection

**Table:**

Bruker D8 Venture Photon 2 diffractometer	3053 reflections with *I* > 2σ(*I*)
Radiation source: Incoatec IµS	*R*_int_ = 0.057
φ and ω scans	θ_max_ = 25.5°, θ_min_ = 2.0°
Absorption correction: multi-scan (SADABS; Bruker, 2016)	*h* = −35→35
*T*_min_ = 0.923, *T*_max_ = 1.000	*k* = −13→13
18758 measured reflections	*l* = −16→16
4007 independent reflections	

### Refinement

**Table:**

Refinement on *F*^2^	Primary atom site location: dual
Least-squares matrix: full	Secondary atom site location: difference Fourier map
*R*[*F*^2^ > 2σ(*F*^2^)] = 0.048	Hydrogen site location: inferred from neighbouring sites
*wR*(*F*^2^) = 0.106	H-atom parameters constrained
*S* = 1.16	*w* = 1/[σ^2^(*F*_o_^2^) + 22.4903*P*] where *P* = (*F*_o_^2^ + 2*F*_c_^2^)/3
4007 reflections	(Δ/σ)_max_ = 0.001
250 parameters	Δρ_max_ = 0.43 e Å^−^^3^
0 restraints	Δρ_min_ = −0.40 e Å^−^^3^

### Special details

**Table:**

Geometry. All esds (except the esd in the dihedral angle between two l.s. planes) are estimated using the full covariance matrix. The cell esds are taken into account individually in the estimation of esds in distances, angles and torsion angles; correlations between esds in cell parameters are only used when they are defined by crystal symmetry. An approximate (isotropic) treatment of cell esds is used for estimating esds involving l.s. planes.

### Fractional atomic coordinates and isotropic or equivalent isotropic displacement parameters (Å^2^)

**Table:**

	*x*	*y*	*z*	*U*_iso_*/*U*_eq_	
Ti1	0.39985 (2)	0.57278 (5)	0.53051 (4)	0.01683 (15)	
Cl1	0.44127 (3)	0.55491 (8)	0.38592 (6)	0.0260 (2)	
Cl2	0.33931 (3)	0.67945 (8)	0.45795 (7)	0.0306 (2)	
O1	0.32753 (8)	0.3882 (2)	0.32835 (17)	0.0271 (6)	
O2	0.27453 (7)	0.4207 (2)	0.44453 (16)	0.0209 (5)	
O3	0.49135 (8)	0.8281 (2)	0.41841 (17)	0.0225 (5)	
O4	0.42031 (7)	0.9091 (2)	0.43313 (16)	0.0189 (5)	
C1	0.34941 (11)	0.4018 (3)	0.4973 (2)	0.0198 (7)	
C2	0.39394 (11)	0.3550 (3)	0.4942 (3)	0.0224 (7)	
H2	0.408032	0.320221	0.438385	0.027*	
C3	0.41406 (12)	0.3689 (3)	0.5889 (3)	0.0249 (8)	
H3	0.444351	0.347138	0.607694	0.030*	
C4	0.38100 (12)	0.4210 (3)	0.6508 (3)	0.0258 (8)	
H4	0.384986	0.438050	0.718950	0.031*	
C5	0.34166 (12)	0.4429 (3)	0.5947 (2)	0.0213 (7)	
H5	0.314378	0.479004	0.617586	0.026*	
C6	0.44378 (11)	0.7612 (3)	0.5464 (2)	0.0178 (7)	
C7	0.47120 (11)	0.6638 (3)	0.5840 (2)	0.0213 (7)	
H7	0.499233	0.636551	0.558028	0.026*	
C8	0.44979 (12)	0.6147 (3)	0.6661 (2)	0.0224 (8)	
H8	0.460880	0.548674	0.705931	0.027*	
C9	0.40869 (12)	0.6805 (3)	0.6796 (2)	0.0230 (7)	
H9	0.387053	0.665144	0.729112	0.028*	
C10	0.40562 (12)	0.7728 (3)	0.6066 (2)	0.0196 (7)	
H10	0.381985	0.832252	0.599266	0.024*	
C11	0.31673 (11)	0.4038 (3)	0.4130 (2)	0.0204 (7)	
C12	0.23511 (11)	0.4317 (3)	0.3749 (2)	0.0225 (7)	
C13	0.24101 (13)	0.5488 (4)	0.3142 (3)	0.0320 (9)	
H13A	0.244351	0.620112	0.358322	0.048*	
H13B	0.214238	0.560587	0.271051	0.048*	
H13C	0.268247	0.541147	0.274311	0.048*	
C14	0.23064 (13)	0.3153 (4)	0.3134 (3)	0.0340 (9)	
H14A	0.255778	0.311161	0.267262	0.051*	
H14B	0.201607	0.316717	0.276553	0.051*	
H14C	0.231637	0.242684	0.356518	0.051*	
C15	0.19555 (12)	0.4444 (4)	0.4438 (3)	0.0360 (10)	
H15A	0.194513	0.371980	0.487035	0.054*	
H15B	0.167056	0.450003	0.405259	0.054*	
H15C	0.199488	0.519323	0.483578	0.054*	
C16	0.45507 (11)	0.8354 (3)	0.4583 (2)	0.0194 (7)	
C17	0.42166 (11)	0.9853 (3)	0.3432 (2)	0.0195 (7)	
C18	0.45974 (11)	1.0803 (3)	0.3530 (3)	0.0229 (7)	
H18A	0.457586	1.122955	0.416323	0.034*	
H18B	0.456899	1.140675	0.299477	0.034*	
H18C	0.489240	1.038412	0.349535	0.034*	
C19	0.42567 (12)	0.9024 (3)	0.2540 (2)	0.0261 (8)	
H19A	0.455422	0.861398	0.255688	0.039*	
H19B	0.422678	0.952354	0.194043	0.039*	
H19C	0.401518	0.839944	0.254504	0.039*	
C20	0.37525 (11)	1.0482 (3)	0.3442 (3)	0.0249 (8)	
H20A	0.351282	0.985237	0.344936	0.037*	
H20B	0.371433	1.099788	0.285353	0.037*	
H20C	0.373206	1.100149	0.403042	0.037*	

### Atomic displacement parameters (Å^2^)

**Table:**

	*U*^11^	*U*^22^	*U*^33^	*U*^12^	*U*^13^	*U*^23^
Ti1	0.0185 (3)	0.0171 (3)	0.0147 (3)	−0.0010 (2)	−0.0016 (2)	0.0002 (2)
Cl1	0.0363 (5)	0.0266 (5)	0.0153 (4)	−0.0001 (4)	0.0043 (4)	−0.0021 (3)
Cl2	0.0258 (5)	0.0207 (4)	0.0448 (6)	−0.0005 (4)	−0.0140 (4)	0.0035 (4)
O1	0.0229 (13)	0.0381 (15)	0.0205 (13)	−0.0010 (11)	0.0017 (11)	−0.0086 (11)
O2	0.0158 (12)	0.0252 (13)	0.0217 (12)	−0.0017 (10)	0.0014 (10)	0.0020 (10)
O3	0.0187 (12)	0.0261 (13)	0.0227 (12)	0.0010 (10)	0.0046 (10)	0.0040 (10)
O4	0.0189 (12)	0.0203 (12)	0.0174 (11)	−0.0005 (9)	0.0000 (9)	0.0033 (9)
C1	0.0230 (17)	0.0133 (16)	0.0230 (17)	−0.0040 (13)	0.0005 (14)	0.0014 (13)
C2	0.0239 (18)	0.0147 (16)	0.0288 (19)	0.0003 (14)	0.0032 (15)	−0.0022 (14)
C3	0.0271 (19)	0.0163 (17)	0.0309 (19)	−0.0023 (14)	−0.0047 (16)	0.0086 (15)
C4	0.031 (2)	0.0252 (19)	0.0208 (17)	−0.0079 (16)	−0.0035 (15)	0.0067 (15)
C5	0.0246 (18)	0.0190 (17)	0.0204 (17)	−0.0033 (14)	0.0014 (14)	0.0055 (14)
C6	0.0196 (17)	0.0192 (17)	0.0145 (15)	−0.0056 (13)	−0.0012 (13)	−0.0012 (13)
C7	0.0165 (17)	0.0283 (19)	0.0187 (17)	−0.0089 (14)	−0.0064 (14)	0.0032 (14)
C8	0.0256 (18)	0.0264 (19)	0.0150 (16)	−0.0097 (15)	−0.0039 (14)	0.0011 (14)
C9	0.0262 (18)	0.0268 (18)	0.0159 (16)	−0.0079 (15)	0.0009 (14)	−0.0040 (14)
C10	0.0256 (18)	0.0169 (17)	0.0164 (16)	−0.0043 (14)	0.0032 (14)	−0.0054 (13)
C11	0.0220 (18)	0.0162 (16)	0.0230 (18)	−0.0051 (14)	0.0015 (15)	−0.0022 (14)
C12	0.0149 (16)	0.0302 (19)	0.0223 (17)	−0.0018 (14)	−0.0017 (14)	−0.0001 (15)
C13	0.025 (2)	0.037 (2)	0.034 (2)	−0.0052 (17)	−0.0070 (17)	0.0057 (18)
C14	0.028 (2)	0.031 (2)	0.043 (2)	−0.0076 (17)	−0.0038 (18)	−0.0079 (18)
C15	0.0198 (19)	0.044 (2)	0.044 (2)	0.0008 (17)	0.0000 (18)	0.000 (2)
C16	0.0194 (17)	0.0187 (17)	0.0200 (17)	−0.0021 (13)	−0.0020 (14)	0.0008 (14)
C17	0.0219 (17)	0.0231 (17)	0.0135 (16)	−0.0003 (14)	0.0012 (14)	0.0057 (13)
C18	0.0211 (17)	0.0216 (18)	0.0258 (18)	−0.0040 (14)	0.0000 (15)	0.0051 (15)
C19	0.0289 (19)	0.031 (2)	0.0183 (17)	−0.0029 (16)	−0.0023 (15)	−0.0005 (15)
C20	0.0214 (18)	0.0267 (19)	0.0265 (19)	0.0006 (15)	0.0018 (15)	0.0053 (15)

### Geometric parameters (Å, º)

**Table:**

Ti1—Cl2	2.3222 (10)	C7—C8	1.397 (5)
Ti1—Cl1	2.3423 (10)	C7—H7	0.9500
Ti1—C9	2.348 (3)	C8—C9	1.417 (5)
Ti1—C8	2.376 (3)	C8—H8	0.9500
Ti1—C3	2.377 (3)	C9—C10	1.409 (5)
Ti1—C4	2.391 (3)	C9—H9	0.9500
Ti1—C5	2.392 (3)	C10—H10	0.9500
Ti1—C10	2.401 (3)	C12—C15	1.514 (5)
Ti1—C1	2.406 (3)	C12—C14	1.516 (5)
Ti1—C2	2.411 (3)	C12—C13	1.523 (5)
Ti1—C7	2.414 (3)	C13—H13A	0.9800
Ti1—C6	2.419 (3)	C13—H13B	0.9800
O1—C11	1.212 (4)	C13—H13C	0.9800
O2—C11	1.333 (4)	C14—H14A	0.9800
O2—C12	1.485 (4)	C14—H14B	0.9800
O3—C16	1.209 (4)	C14—H14C	0.9800
O4—C16	1.334 (4)	C15—H15A	0.9800
O4—C17	1.476 (4)	C15—H15B	0.9800
C1—C2	1.404 (5)	C15—H15C	0.9800
C1—C5	1.420 (5)	C17—C19	1.515 (5)
C1—C11	1.480 (5)	C17—C18	1.522 (4)
C2—C3	1.414 (5)	C17—C20	1.524 (5)
C2—H2	0.9500	C18—H18A	0.9800
C3—C4	1.415 (5)	C18—H18B	0.9800
C3—H3	0.9500	C18—H18C	0.9800
C4—C5	1.392 (5)	C19—H19A	0.9800
C4—H4	0.9500	C19—H19B	0.9800
C5—H5	0.9500	C19—H19C	0.9800
C6—C10	1.407 (5)	C20—H20A	0.9800
C6—C7	1.415 (5)	C20—H20B	0.9800
C6—C16	1.485 (4)	C20—H20C	0.9800
			
Cl2—Ti1—Cl1	95.23 (4)	Ti1—C4—H4	120.6
Cl2—Ti1—C9	100.97 (9)	C4—C5—C1	108.1 (3)
Cl1—Ti1—C9	135.73 (9)	C4—C5—Ti1	73.0 (2)
Cl2—Ti1—C8	133.62 (10)	C1—C5—Ti1	73.32 (19)
Cl1—Ti1—C8	110.13 (9)	C4—C5—H5	126.0
C9—Ti1—C8	34.90 (12)	C1—C5—H5	126.0
Cl2—Ti1—C3	136.89 (9)	Ti1—C5—H5	119.5
Cl1—Ti1—C3	96.52 (10)	C10—C6—C7	108.1 (3)
C9—Ti1—C3	98.96 (12)	C10—C6—C16	128.0 (3)
C8—Ti1—C3	79.42 (12)	C7—C6—C16	123.9 (3)
Cl2—Ti1—C4	116.44 (9)	C10—C6—Ti1	72.34 (18)
Cl1—Ti1—C4	130.53 (10)	C7—C6—Ti1	72.78 (18)
C9—Ti1—C4	76.84 (12)	C16—C6—Ti1	120.7 (2)
C8—Ti1—C4	75.41 (12)	C8—C7—C6	108.0 (3)
C3—Ti1—C4	34.53 (12)	C8—C7—Ti1	71.54 (18)
Cl2—Ti1—C5	84.24 (9)	C6—C7—Ti1	73.17 (18)
Cl1—Ti1—C5	130.25 (9)	C8—C7—H7	126.0
C9—Ti1—C5	92.44 (12)	C6—C7—H7	126.0
C8—Ti1—C5	105.26 (11)	Ti1—C7—H7	121.0
C3—Ti1—C5	57.00 (12)	C7—C8—C9	108.2 (3)
C4—Ti1—C5	33.83 (11)	C7—C8—Ti1	74.55 (18)
Cl2—Ti1—C10	77.43 (9)	C9—C8—Ti1	71.50 (18)
Cl1—Ti1—C10	113.76 (8)	C7—C8—H8	125.9
C9—Ti1—C10	34.50 (11)	C9—C8—H8	125.9
C8—Ti1—C10	57.10 (12)	Ti1—C8—H8	119.9
C3—Ti1—C10	132.89 (12)	C10—C9—C8	107.8 (3)
C4—Ti1—C10	109.79 (12)	C10—C9—Ti1	74.81 (18)
C5—Ti1—C10	114.57 (12)	C8—C9—Ti1	73.60 (19)
Cl2—Ti1—C1	80.71 (8)	C10—C9—H9	126.1
Cl1—Ti1—C1	96.20 (9)	C8—C9—H9	126.1
C9—Ti1—C1	126.83 (12)	Ti1—C9—H9	117.5
C8—Ti1—C1	131.31 (12)	C6—C10—C9	107.9 (3)
C3—Ti1—C1	56.87 (12)	C6—C10—Ti1	73.71 (18)
C4—Ti1—C1	56.65 (11)	C9—C10—Ti1	70.69 (18)
C5—Ti1—C1	34.43 (11)	C6—C10—H10	126.1
C10—Ti1—C1	144.11 (12)	C9—C10—H10	126.1
Cl2—Ti1—C2	110.28 (9)	Ti1—C10—H10	121.3
Cl1—Ti1—C2	77.53 (9)	O1—C11—O2	126.1 (3)
C9—Ti1—C2	131.86 (12)	O1—C11—C1	123.7 (3)
C8—Ti1—C2	112.71 (12)	O2—C11—C1	110.2 (3)
C3—Ti1—C2	34.35 (12)	O2—C12—C15	102.2 (3)
C4—Ti1—C2	56.84 (12)	O2—C12—C14	110.1 (3)
C5—Ti1—C2	56.80 (12)	C15—C12—C14	111.0 (3)
C10—Ti1—C2	166.30 (12)	O2—C12—C13	108.4 (3)
C1—Ti1—C2	33.89 (11)	C15—C12—C13	111.0 (3)
Cl2—Ti1—C7	125.31 (9)	C14—C12—C13	113.6 (3)
Cl1—Ti1—C7	79.83 (9)	C12—C13—H13A	109.5
C9—Ti1—C7	57.19 (12)	C12—C13—H13B	109.5
C8—Ti1—C7	33.92 (11)	H13A—C13—H13B	109.5
C3—Ti1—C7	97.59 (12)	C12—C13—H13C	109.5
C4—Ti1—C7	106.61 (11)	H13A—C13—H13C	109.5
C5—Ti1—C7	138.78 (11)	H13B—C13—H13C	109.5
C10—Ti1—C7	56.64 (12)	C12—C14—H14A	109.5
C1—Ti1—C7	153.80 (12)	C12—C14—H14B	109.5
C2—Ti1—C7	121.21 (12)	H14A—C14—H14B	109.5
Cl2—Ti1—C6	91.27 (8)	C12—C14—H14C	109.5
Cl1—Ti1—C6	81.89 (8)	H14A—C14—H14C	109.5
C9—Ti1—C6	57.05 (11)	H14B—C14—H14C	109.5
C8—Ti1—C6	56.65 (11)	C12—C15—H15A	109.5
C3—Ti1—C6	131.42 (12)	C12—C15—H15B	109.5
C4—Ti1—C6	130.38 (11)	H15A—C15—H15B	109.5
C5—Ti1—C6	147.78 (12)	C12—C15—H15C	109.5
C10—Ti1—C6	33.95 (11)	H15A—C15—H15C	109.5
C1—Ti1—C6	171.57 (11)	H15B—C15—H15C	109.5
C2—Ti1—C6	151.26 (12)	O3—C16—O4	126.9 (3)
C7—Ti1—C6	34.05 (11)	O3—C16—C6	122.8 (3)
C11—O2—C12	121.6 (3)	O4—C16—C6	110.3 (3)
C16—O4—C17	120.8 (3)	O4—C17—C19	109.7 (3)
C2—C1—C5	108.0 (3)	O4—C17—C18	109.7 (3)
C2—C1—C11	124.8 (3)	C19—C17—C18	113.6 (3)
C5—C1—C11	127.2 (3)	O4—C17—C20	101.6 (3)
C2—C1—Ti1	73.25 (19)	C19—C17—C20	110.9 (3)
C5—C1—Ti1	72.25 (18)	C18—C17—C20	110.8 (3)
C11—C1—Ti1	121.4 (2)	C17—C18—H18A	109.5
C1—C2—C3	107.8 (3)	C17—C18—H18B	109.5
C1—C2—Ti1	72.85 (19)	H18A—C18—H18B	109.5
C3—C2—Ti1	71.51 (19)	C17—C18—H18C	109.5
C1—C2—H2	126.1	H18A—C18—H18C	109.5
C3—C2—H2	126.1	H18B—C18—H18C	109.5
Ti1—C2—H2	121.3	C17—C19—H19A	109.5
C2—C3—C4	107.7 (3)	C17—C19—H19B	109.5
C2—C3—Ti1	74.14 (19)	H19A—C19—H19B	109.5
C4—C3—Ti1	73.3 (2)	C17—C19—H19C	109.5
C2—C3—H3	126.1	H19A—C19—H19C	109.5
C4—C3—H3	126.1	H19B—C19—H19C	109.5
Ti1—C3—H3	118.4	C17—C20—H20A	109.5
C5—C4—C3	108.3 (3)	C17—C20—H20B	109.5
C5—C4—Ti1	73.13 (19)	H20A—C20—H20B	109.5
C3—C4—Ti1	72.21 (19)	C17—C20—H20C	109.5
C5—C4—H4	125.8	H20A—C20—H20C	109.5
C3—C4—H4	125.8	H20B—C20—H20C	109.5

### Hydrogen-bond geometry (Å, º)

**Table:**

*D*—H···*A*	*D*—H	H···*A*	*D*···*A*	*D*—H···*A*
C4—H4···Cl1^i^	0.95	2.78	3.632 (4)	149
C7—H7···Cl1^ii^	0.95	2.80	3.511 (4)	132
C8—H8···Cl1^i^	0.95	2.76	3.521 (3)	137
C9—H9···O1^i^	0.95	2.30	3.245 (4)	170

Symmetry codes: (i) *x*, −*y*+1, *z*+1/2; (ii) −*x*+1, −*y*+1, −*z*+1.
